# Commentary: Response of Human Macrophages to Clinically Applied Wound Dressings Loaded With Silver

**DOI:** 10.3389/fbioe.2020.00706

**Published:** 2020-07-14

**Authors:** Linda L. Benskin

**Affiliations:** ^1^Independent Researcher for Wound Care in Remote and Conflict Areas of Developing Countries, Austin, TX, United States; ^2^Ferris Mfg. Corp., Fort Worth, TX, United States

**Keywords:** PolyMem, silver dressings, dressing cytotoxicity, polymeric membrane dressings, inflammation, chronic wounds, macrophages

## Introduction

The authors Varela et al. (2020) pose interesting, relevant questions related to silver-containing wound dressing toxicity, antimicrobial effects, effect on the transition from the M1 to the M2 macrophage phenotypes, and effect on wound inflammation overall. They designed creative studies to answer these questions using six primary dressings: Atrauman, Biatain, and PolyMem WIC, all with and without silver (Varela et al., 2020). Four bacterial strains known to infect open wounds (*Staphylococcus aureus, Staphylococcus epidermidis, Escherichia coli*, and *Pseudomonas aeruginosa*), and three human cell types (fibroblasts, keratinocytes, and macrophages) were cultured. Test dressing discs were placed over two-dimensional bacteria and cell culture colonies, which were then incubated (Varela et al., 2020). Wells with test dressings covered with 2 mls of a bacterial broth were also incubated (Varela et al., 2020). The descriptions of wound healing, materials used, tests, and rationale are clear (Varela et al., 2020).

However, the study results are unlikely to be clinically meaningful because of two major design flaws: (1) Contrary to the Instructions for Use, the researchers did not provide secondary (cover) dressings to prevent desiccation with any of the six study dressings (Hess, 2020; Atrauman, 2020), and (2) The *in vitro* techniques used did not capture the multifunctional interactive attributes of polymeric membrane dressings (PMDs) such as PolyMem WIC and PolyMem WIC Silver, rending the results for these dressings clinically irrelevant (Benskin, 2012, 2016a).

## Antibacterial and Cytotoxicity Tests for All Test Dressings Confounded by Desiccation

All six test dressings are designed to be covered with a secondary dressing to maintain appropriate wound moisture (Hess, 2020; Atrauman, 2020). Atrauman (with and without silver) is intended to keep relatively dry wounds moist, making it somewhat suitable for testing on agar (Atrauman, 2020). In contrast, PolyMem WIC and Biatain (with and without silver) are designed for cavity and heavily exudating wounds, making them unsuitable (Hess, 2020). Because all six dressings were tested without moisture-retentive coverings (Varela et al., 2020), the probability that many of the plated bacterial and human cells died due to desiccation, rather than due to the toxicity of any particular dressing components, cannot be discounted.

PolyMem WIC dressings gradually release glycerol, which draws fluid from the body into the wound bed (Cutting et al., 2015; Benskin, 2016b, 2018). From there, the fluid is absorbed by the superabsorbent within the dressings and the dressing substrate itself (Cutting et al., 2015; Benskin, 2016b, 2018). Without a body to donate fluid, both the standard and the silver PolyMem WIC entrapped a part of the agar in the dressing, providing evidence of desiccation and a failure of the experimental model (Benskin, 2016a,b; Varela et al., 2020). Desiccation as an unmeasured confounding variable would explain why Atrauman, with and without silver, the test dressings with the greatest ability to preserve wound moisture *in vitro* without a cover dressing, appeared to be less cytotoxic than the other test dressings, including the other two without silver (Atrauman, 2020; Varela et al., 2020).

## Cytotoxicity Test of Polymem Dressings Confounded by Non-Interactive *in vitro* Setting

As the authors noted, rather than distributing cytotoxic silver into the wound bed like the other silver test dressings, PolyMem Silver's continuous wound cleansing system interacts with the body to dislodge wound pathogens and pull them into contact with silver locked in the dressing (Burd et al., 2007; Benskin, 2012, 2018; Varela et al., 2020). Rather, *in vitro*, PolyMem WIC had no body from which to draw new moisture to replace what was absorbed, impairing the ordinarily robust moisture-balancing system and leading to cell death by desiccation (Benskin, 2016a, 2018).

## Antibacterial Tests of Polymem Dressings Confounded by Non-Interactive *in vitro* Setting

PolyMem Silver cannot demonstrate its antibacterial benefits on a petri dish because virtually all of the silver is retained safely in the dressing substrate (Burd et al., 2007). Failure to take PolyMem's powerful continuous wound cleansing system into account also confounded the antibacterial tests. In the disc diffusion test, the entrapped agar is evidence of this study design failure (Varela et al., 2020). In the broth medium experiment, after PolyMem WIC Silver was completely saturated, bacteria remaining in the broth could not be pulled into contact with the silver in the PolyMem (Varela et al., 2020).

## Wound Inflammation Tests of Polymem Dressings Confounded by Non-Interactive *in vitro* Setting

The statement that PolyMem is “defined” as an “anti-inflammatory dressing” is a gross over-simplification (Varela et al., 2020). Controlled inflammation is beneficial throughout the wound healing process (Davies and White, 2011). PolyMem dressings (with and without silver) focus inflammation, permitting the robust concentration of macrophages and neutrophils at the injury site that is necessary for brisk healing to take place, while virtually eliminating the secondary inflammation that causes poor perfusion (Beitz et al., 2004; Davies and White, 2011; Benskin, 2012; Weissman et al., 2013). This highly desirable alteration in the inflammatory response is not attributable to any individual component, such as the silver, glycerol, surfactant, or superabsorbent, but rather, it is the synergistic effect of PolyMem's multiple components interacting with the body's nociceptor response (Kahn et al., 2000; Beitz et al., 2004; Weissman et al., 2013; Benskin, 2016a).

**Figure 1:**
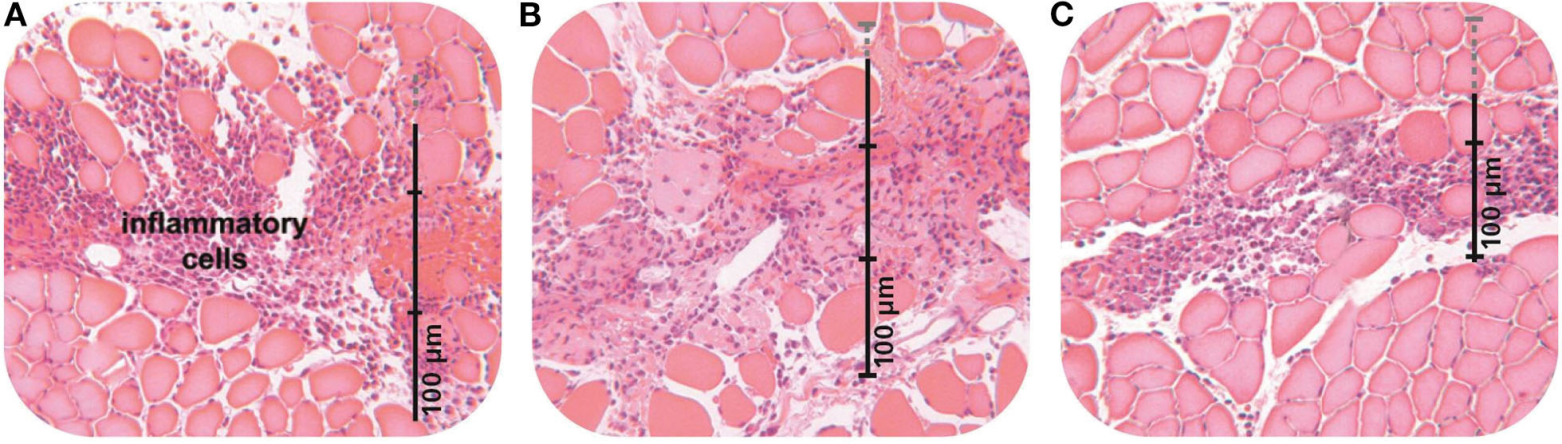
The vertical lines are rulers measuring the spread of the inflammatory cells (purple) in the muscle tissue (pink). The overall number of WBCs is reduced with both the conventional dressing **(B)** and PolyMem **(C)** when compared with no dressing **(A)**. However, in contrast to the conventional dressing, which does not limit the spread of the inflammatory cells when compared with no dressing at all, PolyMem directs the inflammatory cells into the incision area, where they are needed, while dramatically decreasing the number of inflammatory cells in the surrounding tissues, where they would be destructive (from Beitz et al., 2004).

Varela et al. measured the total number and phenotypes of the macrophages and inflammatory markers, failing to examine their location within the wound (Varela et al., 2020). Beitz et al. applied PolyMem dressings to a stab wound, finding that the inflammatory cells deep in the muscle tissue lined up along the incision line, with almost no inflammatory cells in the surrounding tissue (see Figure 1) (Beitz et al., 2004). And, applying PolyMem to distal open or closed tissue injuries altered inflammation markers in the spinal cord laminae (Beitz et al., 2004). Kahn et al., found that bruising and edema were dramatically reduced when PolyMem was applied to intact skin immediately following deep tissue mechanical trauma (Kahn et al., 2000). Varela et al. could not measure these critical distinctions between PolyMem and other wound dressings with respect to inflammation because *in vitro* models cannot include an intact central nervous system (Benskin, 2018; Varela et al., 2020).

## Discussion

The absence of secondary dressings in this study design would result in varying degrees of cell desiccation, a confounding variable that could have influenced bacterial and human cell death quite apart from the effects of the silver in the test dressings (Hess, 2020; Atrauman, 2020). This renders the results of the bactericidal and cytotoxicity aspects of this study clinically irrelevant. And, testing any configuration of PMDs *in vitro* (or *ex vivo*) will fail to capture all the benefits of PolyMem dressings which are achieved through their complex synergistic interaction with the patient's body, such as controlling and focusing inflammation, continuously cleansing wounds, and balancing moisture (Weissman et al., 2013; Benskin, 2016a,b; Benskin, 2018).

Perhaps new devices will be invented to allow non-invasive dynamic wound inflammation evaluation, similar to current oxygen saturation monitors. Until these are available, inflammatory markers in the exudate from PolyMem and other spent dressings could be compared.

## Author Contributions

The author confirms being the sole contributor of this work and has approved it for publication.

## Conflict of Interest

LB is an employee of Ferris Mfg. Corp., makers of PolyMem and PolyMem Silver dressings.
